# A New SAR Image Segmentation Algorithm for the Detection of Target and Shadow Regions

**DOI:** 10.1038/srep38596

**Published:** 2016-12-07

**Authors:** Shiqi Huang, Wenzhun Huang, Ting Zhang

**Affiliations:** 1 Department of Electronic Information Engineering, Xijing University, Xi’an, 710123, China

## Abstract

The most distinctive characteristic of synthetic aperture radar (SAR) is that it can acquire data under all weather conditions and at all times. However, its coherent imaging mechanism introduces a great deal of speckle noise into SAR images, which makes the segmentation of target and shadow regions in SAR images very difficult. This paper proposes a new SAR image segmentation method based on wavelet decomposition and a constant false alarm rate (WD-CFAR). The WD-CFAR algorithm not only is insensitive to the speckle noise in SAR images but also can segment target and shadow regions simultaneously, and it is also able to effectively segment SAR images with a low signal-to-clutter ratio (SCR). Experiments were performed to assess the performance of the new algorithm on various SAR images. The experimental results show that the proposed method is effective and feasible and possesses good characteristics for general application.

Image segmentation is fundamental to the fields of image analysis, image understanding, and image pattern recognition. It is also an important part of image processing. For this reason, image segmentation has been widely used in many fields, such as biomedicine, remote sensing, video communication, public security, transportation, agriculture, environmental analysis, ecology, geology, weather prediction, disaster assessment, and search and rescue^1^,^2^,^3^. With the continuing development of image segmentation technology and theory, its range of practical applications may further increase. Although an image can provide a large amount of useful information, only some parts of each image are relevant, whereas others are not. The region of interest (ROI) in an image is often called the target region, and other regions are called background regions. Image segmentation is the process of partitioning an entire image into disjoint and homogeneous regions based on certain criteria. Image segmentation divides an image into several parts. Each part is an independent, physically meaningful area that acts as a unit. These individual regions do not overlap each other, and although the information contained in each segmented region has similar characteristics, these characteristics differ across different segmented regions.

The theory and methods of image segmentation are very well developed. To date, more than one thousand image segmentation methods (algorithms) have been proposed, and new image segmentation algorithms are still being continuously introduced^4^,^5^,^6^,^7^,^8^,^9^,^10^,^11^,^12^. Although there are various types of image segmentation algorithms, each algorithm is usually tailored to a certain application background before it is presented. There is currently no single theory or method, no universal segmentation framework that can be used on any and all images. There is also no uniform standard for the assessment of segmentation results, and this process still largely relies on visual analysis and personal judgment. In general, image segmentation methods can be divided into four categories: (1) threshold-based image segmentation, (2) edge-detection-based image segmentation, (3) region-growth-based image segmentation, and (4) specific-theory-based image segmentation. Threshold segmentation involves the use of image gray values to segment images. It encompasses both single-threshold segmentation and multi-threshold segmentation. The advantage of threshold segmentation is that it is simple and easy to perform, and its disadvantage is that it is suitable only for images with strong contrast between the target and background areas. Edge detection involves detecting and connecting edge points to produce a sub-image with boundaries that segment the image of interest. Methods of edge detection segmentation can be divided into groups based on the techniques used for edge detection, such as serial edge detection segmentation and parallel edge detection segmentation. Region segmentation is usually performed based on statistical features, which are used to divide an image into different subregions. Typical methods for region segmentation include the region-growing method, the split-and-merge method, and the watershed method. Improvements in science and technology have allowed users to combine image segmentation with a wide variety of specific theories and technologies, which has led to many new theories and methods of image segmentation^13^,^14^,^15^. These new methods have produced very good segmentation results.

Synthetic aperture radar (SAR) is a form of active microwave imaging radar. Its most distinctive characteristic is that it can be used to collect remote sensing data under all weather conditions and at all times; consequently, it has been widely used in both civilian and military applications. However, its coherent imaging mechanism produces images that always contain a large amount of speckle noise, which makes the segmentation and use of SAR images very challenging. When an image segmentation algorithm is directly applied to segment a SAR image, it is very difficult to achieve an ideal segmentation effect. For this reason, many scholars have used the characteristics of SAR images and their specific application backgrounds to develop many different segmentation algorithms that are tailored particularly for SAR images^16^,^17^,^18^,^19^. These algorithms can be divided into two categories based on the purpose of segmentation. One category consists of image segmentation methods meant to extract a target of interest; the other consists of image segmentation methods designed to classify ground objects. In terms of their approaches to image segmentation and reducing the amount of speckle noise in SAR images, SAR image segmentation methods can be divided into direct image segmentation methods and post-filtered image segmentation methods. In direct SAR image segmentation, which generally involves the statistical modeling of SAR data, the removal of speckle noise is considered in the segmentation model. Representative algorithms include image segmentation based on a constant false alarm rate (CFAR)^20^, image segmentation based on Markov random field (MRF)^21^,^22^ and image segmentation based on edge detection^23^.

Image segmentation algorithms based on CFAR detection function as follows. Initially, each image’s statistical characteristics are estimated, and a threshold is determined. Then, the gray level value of each pixel in the image is compared against the threshold value. Finally, the image is segmented. The advantage of the CFAR-detection-based segmentation algorithm is its fast segmentation speed; its disadvantage is that it considers only the gray information in the image and does not consider spatial information, and consequently, the segmentation results often contain speckle noise, which renders this approach impractical. Although the MRF-based SAR image segmentation algorithm considers the spatial neighborhood structure of every pixel, its shortcomings are also considerable. For example, the amount of data to be processed is large, the convergence speed is slow, many parameters need to be adjusted, and it is very difficult to optimize. Meanwhile, the edge-detection-based SAR image segmentation algorithm can be profoundly affected by speckle noise. When there is a large amount of speckle noise in a SAR image, it is often relatively difficult for the edge detection operators to produce good edge maps, which makes it difficult to accurately estimate the positions of the edge pixels.

Based on the characteristics of SAR images and using CFAR detectors and multi-scale wavelet decomposition theory, this paper proposes a new kind of SAR image segmentation algorithm based on wavelet decomposition and the CFAR approach, called the WD-CFAR algorithm. The algorithm first performs wavelet decomposition on the SAR image; next, it selects several high-frequency coefficient sub-images for CFAR detection and then performs an inverse wavelet transform and subtracts the mean value from the image; and finally, it performs a second round of CFAR detection to identify the target and shadow regions in the SAR image. The WD-CFAR algorithm has the following advantages: (1) it is not sensitive to speckle noise, i.e., it reduces the influence of speckle noise on SAR image segmentation; (2) unlike single CFAR detectors, it does not require high contrast between the target and background; (3) it can detect the target and shadow regions simultaneously; (4) it can segment weak scattering targets in SAR images, such as runways and highways; and (5) it offers strong adaptability for use with different types of SAR images, especially those of low and moderate resolution. The current paper makes several contributions to the field. The first is the conceptual design for two-tiered CFAR detection; the second is the combination of multi-scale wavelet decomposition with CFAR detectors; the third is the improvement in segmentation performance achieved through the subtraction of the mean value; and the fourth is the provision of an avenue for the segmentation of target and shadow regions of different reflection intensities. Tests of the WD-CFAR algorithm on practical SAR image data have shown it to be an effective and feasible segmentation algorithm.

## CFAR detection algorithm and wavelet decomposition theory

### CFAR detection algorithm

CFAR detection is a common method of radar target detection. It involves estimating a detection threshold based on the background against which a target is to be detected. Because of its simple calculation, constant rate of false positives (false alarms), adaptive threshold adjustment, and rapid detection, the CFAR approach has been widely studied and applied for target detection and segmentation in SAR images^24^,^25^. In SAR images, a target is often located in a complex background environment. This is especially true of certain small targets, weakly scattering targets, and concealed targets. If only a fixed threshold is used for target detection, it is very difficult to achieve ideal performance. For this reason, most forms of SAR target detection require an adaptive threshold detector. The CFAR detector is appropriate for automatic methods of threshold detection based on pixel levels, in which adaptive threshold selection and determination are performed relative to the statistical distribution model of the background region in which the target is located and the rate of false alarms is constant. If the rate of false alarms is known, then analysis of the statistical distribution characteristics of a SAR image can be used to determine the detection threshold for target detection or segmentation. The CFAR detector usually requires there to be a strong contrast between the target and background regions. In the case presented here, it has been found to perform better than other methods. The WD-CFAR algorithm proposed in this paper combines two-tiered CFAR detection with wavelet decomposition to overcome this disadvantage of CFAR detection.

The key to SAR target detection based on the CFAR approach is the determination of the detection threshold. It is an adaptive detection technology, and when the background is known and the rate of false alarms is constant, its performance depends on the chosen threshold. There is a very close relationship between the determination of the threshold *T* and the background clutter distribution model of the SAR image of image. Let the probability density functions for the background and target regions in the SAR image be denoted by *p*_B_(*x*) and *p*_T_(*x*), respectively. Then, the definitions of the false alarm rate *P*_f_ and the detection rate *P*_d_ are given by Equations (1) and (2), respectively.


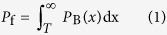



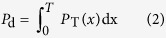


The threshold *T* can be determined by solving Equation (3).


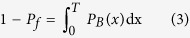


Here, *P*_f_ denotes the probability of false alarms. Different values of *P*_f_ can produce different values of *T*. The possible types of clutter distributions in SAR images mainly include the lognormal, Rayleigh, Weibull, K, Gamma, and Pearson distributions. The Rayleigh distribution is a special case of the Weibull distribution. The detection rule is as follows.


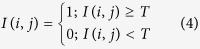


Here, *I*(*i, j*) denotes the gray value of the pixel at point (*i, j*); a value of 1 indicates a target region, and a value of 0 indicates a background region.

### Wavelet decomposition theory

**Figure 1:**
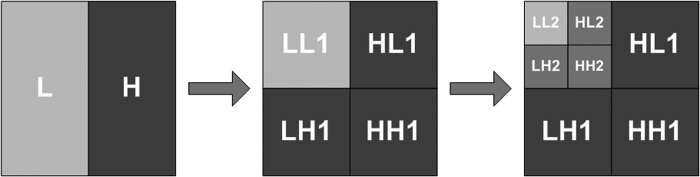
Schematic illustration of wavelet transformations performed on an image.

Using the multi-scale wavelet technique to decompose an image produces a pyramid structure of decomposition coefficients. When an image is decomposed using a two-dimensional discrete wavelet transform, each decomposition operation produces one low-frequency sub-image (LL) and three high-frequency sub-images, i.e., a horizontal sub-image (LH), a vertical sub-image (HL), and a diagonal sub-image (HH). The next level of wavelet decomposition is then applied to the low-frequency sub-image LL that is obtained via decomposition at the previous level. Similarly, this process also produces one low-frequency sub-image and three high-frequency sub-images. This process is repeated until the decomposition reaches level *N*, which is the number of pre-set decomposition levels (scales). Sub-images obtained at low scales reflect high-frequency information, and sub-images obtained at high scales reflect low-frequency information. The highest scale contains more than 95% of the energy, but the majority of it is background and trend information. The process of two-dimensional wavelet decomposition is illustrated in Fig. 1.

Figure 1 shows a decomposition process with two scales. As shown in Fig. 1, 3*N* + 1 sub-images have been produced once an image has been decomposed at level *N*. Furthermore, with an increment of one in the decomposition scale, the size of the corresponding sub-images is 1/2 that of the sub-images at the next higher scale. Specifically, two-dimensional discrete wavelet decomposition is a process of down-sampling by two. In the WD-CFAR algorithm, however, instead of a two-dimensional discrete wavelet transform (DWT), a two-dimensional stationary wavelet transform (SWT) is used, which does not perform down-sampling by two. Therefore, the sub-images obtained using the two-dimensional SWT are of the same size as the original image.

The most important step in two-dimensional wavelet decomposition is to determine the number of decomposition scales, *N*. If this number is too low, full use cannot be made of the advantages of wavelet decomposition. If the number of scales is too high, the decomposition coefficients obtained at higher scales cannot yield any new low-frequency information. The most suitable decomposition scale is called the optimal scale, which is represented here by the variable *r*. The optimal scale can be determined by considering that boundary and homogeneous regions consist of pixels at different scales. At a low resolution, the pixels in a boundary or homogenous region can be used to determine whether any other pixel in that region is unreliable. At these decomposition scales, the detail and edge information can be ignored in the decomposition process. For some pixels, however, not only the edge information but also the geometric details are needed to determine whether the scale of that pixel is stable or optimal.

At a given scale *n*, the multi-scale local coefficient of variation (*LCV*_n_) is used to determine whether a given pixel belongs to an edge region or a homogenous region^26^. The coefficient of variation can be used to index and describe the degree of local non-homogeneity in SAR images; it also serves as a theoretical value that reflects the intensity of speckle noise. The definition of *LCV*_n_ is given as follows.


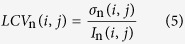


Here, σ_n_(*i, j*) and *I*_n_(*i, j*) represent the local standard deviation and mean, respectively. Equation (5) is used to calculate the local coefficient of variation at spatial position (*i, j*) at decomposition scale *n*, where *n = 1, 2, ···, N − 1, N* and *N* represents the highest scale of the wavelet decomposition.

To improve the accuracy of the calculation, a sliding window is used to compute *LCV*_n_, and the size of that sliding window is determined by the user. If the sliding window is too small, the local statistical parameters become less reliable; if the sliding window is too large, the sensitivity to geometric details decreases. Therefore, the size of the sliding window should be chosen by considering the balance between these two properties. Experiments have shown that a sliding window with dimensions of 5 × 5 or 7 × 7 is generally suitable. The relationship between the sliding window size and the optimal decomposition scale is discussed in detail in the experimental sections. The coefficient of variation is an estimate of the heterogeneity of the scene; a low value indicates a homogeneous region, and a high value indicates a non-homogeneous region (such as an edge or point target). To judge between similar regions and heterogeneous regions, a threshold must be defined. At decomposition scale *n*, the global coefficient of variation (*GCV*_n_) represents the homogeneity of similar regions; it is defined as follows.


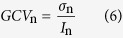


Here, σ_n_ and *I*_n_ denote the standard variance and mean of the coefficient sub-image at decomposition scale *n*. At a given scale, regions can be identified as similar when their coefficients of variation satisfy the following condition.


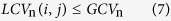


If all pixels satisfy the condition given in Equation (7) at a decomposition scale *n*, then this decomposition scale *n* can be considered the optimal scale *r (r = n,* where *n* = 1, 2, …, *N − 1, N*).

## Principle of the WD-CFAR algorithm

SAR imaging is the process of mapping from scattering feature space to image space. During this process, speckle noise is introduced that affects SAR image segmentation, but radar imaging system parameters (incidence angle, polarization, frequency) and ground surface parameters (roughness, dielectric constant) can also affect SAR image segmentation. For this reason, the fact that different targets in SAR images usually reflect different amounts of light becomes relevant. For example, airport runways and still water are dark in SAR images, whereas other man-made objects, such as vehicles and houses, are bright. These factors hinder SAR target and shadow segmentation.

A SAR image that contains small, weakly scattering and concealed targets usually has a low signal-to-clutter ratio (SCR) or signal-to-noise ratio (SNR), and it is very difficult to completely segment these target regions using conventional methods.

**Figure 2:**
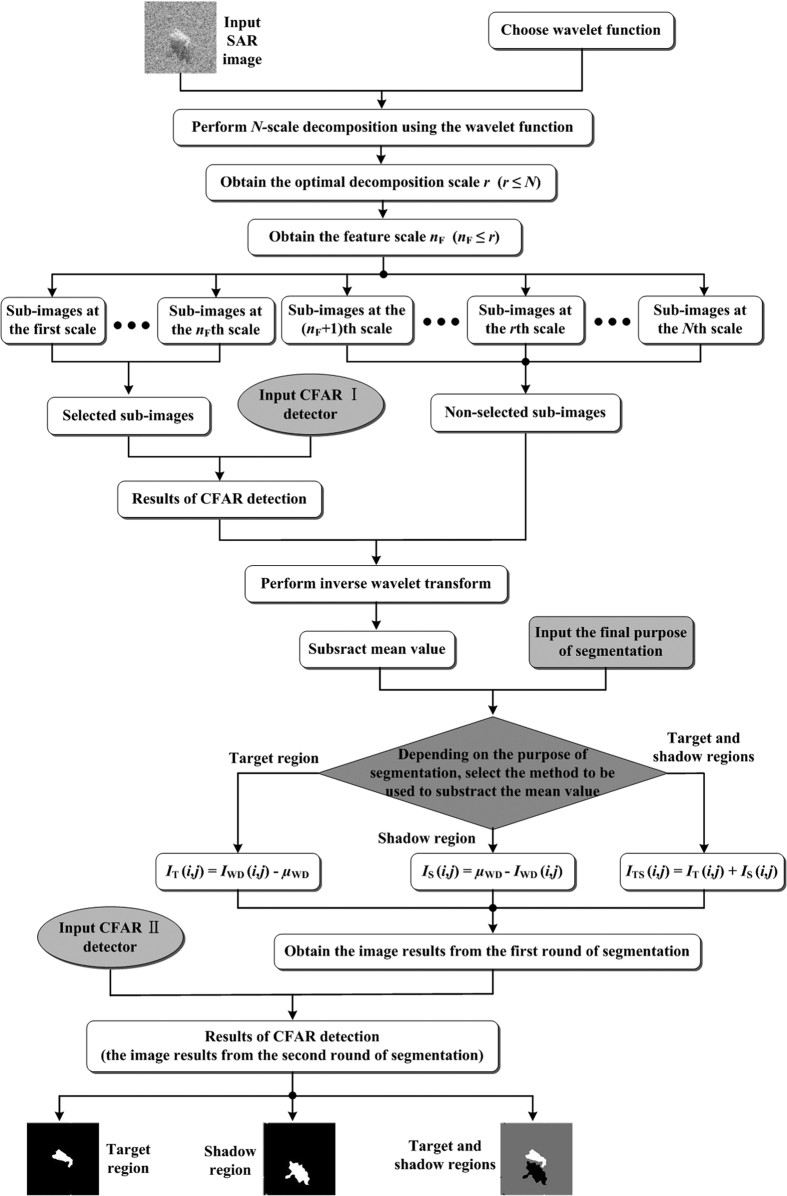
Flowchart of the WD-CFAR algorithm.

The WD-CFAR algorithm proposed in this paper uses two-tiered CFAR detection to overcome the obstacle of low contrast between the target and background regions, and it also incorporates multi-scale wavelet decomposition to its sensitivity to speckle noise. A flowchart of the WD-CFAR algorithm is shown in Fig. 2, and the steps of the algorithm are as follows.

1. Input the SAR image. The original SAR image is entered into the system for segmentation. Notably, the SAR image segmentation process discussed herein is focused on target region segmentation rather than ground target classification. If there is a shadow region and it is part of the ROI, then that region will be segmented. Therefore, the target or shadow region of interest occupies only part of the entire SAR image.
2. Select the wavelet function. There are many types of wavelet functions, and different wavelet functions produce different results because of the differences in their structures. The Daubechies (db*M*) wavelet family has a compact support set, and its members are orthogonal and regular. Their regularity increases as the sequence number *M* increases. The Daubechies 4 (db4) wavelet function not only exhibits better denoising performance but also involves simpler decomposition and reconstruction of the filter coefficients. For this reason, the db4 wavelet function is preferred for the decomposition of SAR images using the WD-CFAR algorithm.
3. Perform multi-scale wavelet decomposition. The input SAR image is decomposed via a multi-scale decomposition operation using the wavelet function selected in step (2), and several sub-images are obtained at different scales. The most important step is to determine the optimal decomposition scale *r*, as presented in the previous section. *N* is the total number of decomposition scales. Simultaneously, the two-dimensional SWT is applied to decompose the SAR image.
4. Select the coefficient sub-images to be used in the first-round CFAR process. When the SAR image is subjected to wavelet decomposition, if the decomposition scale is *N*, the process will produce 3 *N* + 1 coefficient sub-images. In the first round of CFAR detection, not all of these sub-images are subjected to CFAR detection; instead, only some of the sub-images are selected for detection. As the decomposition scale increases, the sub-images contain fewer high-frequency components, and thus, the noise and target information is collected mainly in the low-scale sub-images; high-scale sub-images mainly contain background clutter information. For this reason, the WD-CFAR algorithm selects only low-scale coefficient sub-images on which to perform CFAR detection segmentation. The selected scale is called the feature scale, and it is denoted by *n*_F_, *n*_F_ ≤ *r*. In the WD-CFAR algorithm, *n*_F_ = 2. Thus, the coefficient sub-images at the first and second scales are selected, and these selected sub-images are used to perform the first round of CFAR detection segmentation. The selection principle for the feature scale *n*_F_ is described in detail in the experimental sections.
5. Input the CFAR I detector. The purpose of this step is to set the false alarm rate *P*_f_, which is constant. Different false alarm rates can affect the SAR image segmentation results. Different SAR images may also yield different rates of false alarms.
6. Perform detection operations on the selected sub-images using the CFAR I detector. Using the previously set false alarm rate and the selected coefficient sub-images, CFAR detection segmentation is performed to obtain the first round of segmentation results.
7. Perform the inverse wavelet transform. All decomposed wavelet coefficient sub-images, including both the sub-images that were selected for CFAR detection processing and those that were not selected, are subjected to reconstruction operations, i.e., the inverse wavelet transform. This produces a new SAR image following the first round of detection with the CFAR I detector.
8. Input the final purpose of SAR image segmentation. When segmenting a SAR image that includes man-made objects, there are three modes that can be chosen. The first is the segmentation of the target region, the second is the segmentation of the shadow cast by the target, and the third is the segmentation of both, i.e., the segmentation of the target and shadow regions.
9. Subtract the mean value. The SAR image obtained after CFAR detection is subjected to the mean value removal operation. In the previous steps of the workflow, no processing has been performed on the low-frequency components of the SAR image because they include only background clutter information, which is not the goal of segmentation. Essentially, the subtraction of the mean value is equivalent to the removal of background clutter components to improve performance in the second round of segmentation processing using the CFAR II detector. Simultaneously, the operation also removes much of the speckle noise introduced by the SAR imaging mechanism.The method used to subtract the mean value differs depending on the specific purpose of the current segmentation process. This can affect the final segmentation results. If the components of the SAR image to be extracted or segmented include the target region, then Equation (8) is used for mean value subtraction.
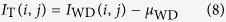
Here, *I*_WD_(*i, j*) denotes the SAR image that has been processed via wavelet decomposition, *μ*_WD_ is the mean value of *I*_WD_(*i, j*), and *I*_T_(*i, j*) is the image of the target region after the mean value has been subtracted from *I*_WD_(*i, j*).If the purpose is to segment the shadow region, then the mean value subtraction is performed as follows.
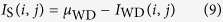
In Equation (9), *I*_S_(*i, j*) is the shadow region created by the man-made target in the SAR image.If the regions to be segmented include both the target and shadow regions in the SAR image, the mean value subtraction operation is performed using Equation (10).
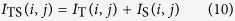
Here, *I*_TS_(*i, j*) represents the region consisting of the target and its shadow, which are segmented simultaneously. Note that *I*_T_(*i, j*) and *I*_S_(*i, j*) are computed using Equations (8) and (9), respectively. That is, Equation (10) cannot be calculated through direct addition operations as Equations (8) and (9) are. These calculations must be performed separately.
10. Obtain the first-round segmentation image *Î*_seg_(*i, j*). After the processing described above, the image will contain mainly target (or shadow) region components. However, it will also include some background information, so it must be subjected to a further segmentation process.
11. Input the CFAR II detector. As in the case of the CFAR I detector, the key step is to set the false alarm rate; however, this value will be lower for the CFAR II detector than for the CFAR I detector.
12. Perform the second round of CFAR detection and segmentation processing. The application of the CFAR II detector completes the second round of detection and segmentation for the SAR image. After this round of processing, the final segmented version of the SAR image, *I*_seg_(*i, j*), is produced.

## Experimental results and analysis

### Segmentation experiments on various SAR images using different algorithms

**Figure 3:**
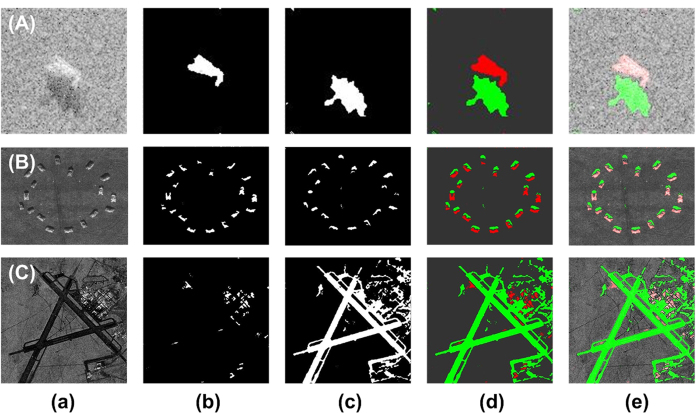
Segmentation results for various SAR images using the WD-CFAR algorithm. ((**a**) Original SAR images, (**b**) Target region segmentation, (**c**) Shadow region segmentation, (**d**) False-color representation of results, (**e**) Combination of segmentation results and original images.)

To demonstrate and confirm the feasibility of the WD-CFAR algorithm, several comparative experiments were performed. The first experiment addressed SAR image segmentation using the WD-CFAR algorithm, and the experimental results are shown in Fig. 3. Figure 3(a) shows the various original SAR images, labeled as Fig. 3(A–C). The SAR image data presented in Fig. 3(A) were obtained from the public MSTAR (Moving and Stationary Target Acquisition and Recognition) database. The target region contains several tanks, and the image size is 128 × 128. The SAR image data shown in Fig. 3(B,C) were obtained from the open image database of Sandia National Laboratories. The image presented in Fig. 3(B) contains various military vehicles and tanks, and its size is 512 × 400. The image presented in Fig. 3(C) shows an airport runway target, and its size is 400 × 400. For the image in Fig. 3(C), the purpose of segmentation was to segment the airport runway and airport buildings; however, the runway is a weakly scattering target, so in this experiment, it was treated as a shadow region for processing. These SAR images share two common characteristics. First, they are all airborne SAR images; second, they are low-SNR or low-SCR images.

The SNR of an image is defined as the ratio between the power spectra of the signal and the noise. It is very difficult to compute these power spectra. However, the ratio of the signal variance to the noise variance can usually be used to approximate the value of the SNR. The local variance of all pixels in the image is computed; the maximum local variance is taken as the signal variance, and the minimum value is taken as the noise variance. The ratio of the maximum variance to the minimum variance is taken as the SNR, which is then converted to a value in units of dB. The approximate mathematical model for computing the SNR is as follows.


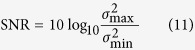


Here, *σ*^*2*^_max_ is the maximum variance value and *σ*^*2*^_min_ is the minimum variance value. To compute the local variances, a sliding window must be established. The size of the sliding window has little effect on the SNR value. For this reason, in the WD-CFAR algorithm, the size of the sliding window used to compute the SNR is set to 100 × 100. If the SNR value is greater than or equal to 100, i.e., 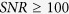, then the image is considered to be a high-SNR image; if *SNR* < 100, then it is a low-SNR image. All SNR values for Fig. 3(A–C) are shown in Table 1.

Figure 3(b) shows the results of target region segmentation. The segmentation results show that the WD-CFAR algorithm can effectively segment target regions from low-SNR SAR images. Here, the airport runway shown in Fig. 3(C) is used as an example of shadow processing. Figure 3(c) shows the results of shadow region segmentation. The segmentation effect is very good, and the results show that the WD-CFAR algorithm can be effectively used to perform segmentation and extract shadow regions in the case of images with a low SNR. False-color representations of the segmentation results are shown in Fig. 3(d,e), where red indicates a target region and green indicates a shadow region. These experimental results show that the segmentation of SAR images using the WD-CFAR algorithm is feasible. All parameters used in the above experiments are presented in Table 1.

To demonstrate the feasibility and effectiveness of the WD-CFAR algorithm, comparative experiments were conducted using different segmentation algorithms. The algorithms considered for comparison included the direct CFAR detection algorithm, the direct wavelet transform algorithm, and the MRF segmentation method. The main difference between the direct wavelet transform algorithm and WD-CFAR algorithm is that the direct wavelet transform algorithm performs detection and segmentation on all sub-images and then applies the inverse wavelet transform, whereas the WD-CFAR algorithm performs detection and processing on only certain selected coefficient sub-images, without applying any processing to the other sub-images that are not selected, before finally subjecting all sub-images (including both the selected and non-selected coefficient images) to the inverse wavelet transform. Moreover, neither the direct CFAR algorithm nor the wavelet transform algorithm performs mean value subtraction. The subtraction of the mean value is a prominent advantage and innovative feature of the WD-CFAR algorithm.

**Figure 4:**
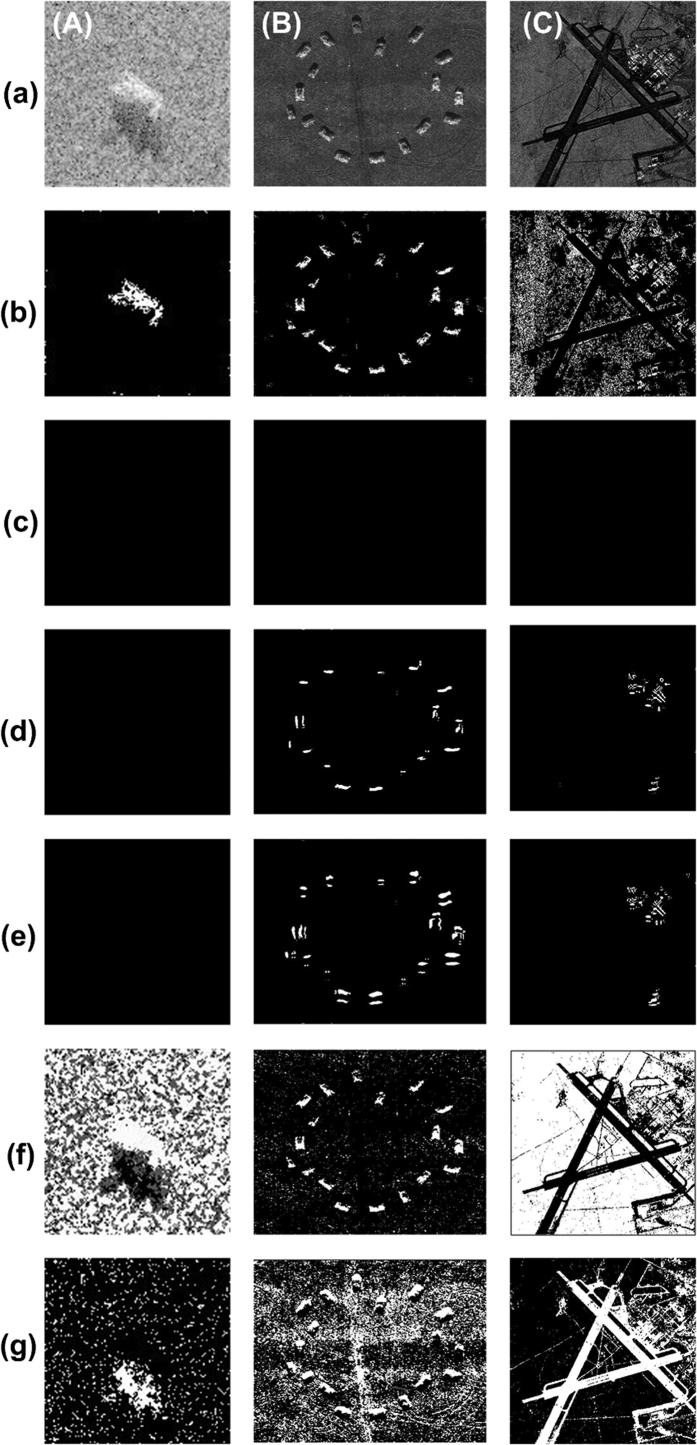
Segmentation results obtained using the direct CFAR algorithm, the direct wavelet transform algorithm and the MRF algorithm. ((**a**) Original SAR images, (**b**,**c**) is target and shadow region segmentation with CFAR method respectively, (**d,e**) is target and shadow region segmentation with wavelet transform respectively, (**f,g**) is target and shadow region segmentation with MRG method respectively.)

The experimental data and the corresponding parameters were identical to those used in the experiments presented in Fig. 3, and the experimental process was also the same. The experimental results are shown in Fig. 4. Here, Fig. 4(a) shows the original images. Figures 4(A–C) are identical to Fig. 3(A–C). Figure 4(b) shows the results of target region segmentation obtained via two rounds of direct CFAR detection. It is obvious that the effect is not as good as that of the WD-CFAR algorithm, especially for the target region detection for Fig. 4(C). Regarding shadow region segmentation, the CFAR detection algorithm cannot successfully perform this task, as shown in Fig. 4(c), which indicates that no shadow information was detected. Figure 4(d,e) show the experimental segmentation results achieved using only multi-scale wavelet decomposition theory; they show the results of target and shadow region segmentation, respectively. When multi-scale wavelet decomposition theory alone is used to segment SAR image targets, especially in low-SNR images, it is very difficult to achieve ideal segmentation results. The WD-CFAR algorithm is not a simple combination of the CFAR algorithm and multi-scale wavelet decomposition theory. By using the characteristics of SAR images and the SAR imaging mechanism, the WD-CFAR algorithm makes full use of the advantages of both the CFAR principle and multi-scale wavelet decomposition and overcomes their shortcomings to achieve effective target segmentation. For example, simple CFAR detection is suitable only for high-contrast images, and in the basic wavelet algorithm, all decomposed coefficient sub-images are used blindly for detection, such that the effects of background clutter cannot be eliminated. The segmentation results obtained using the MRF algorithm are shown in Fig. 4(f,g), where the former shows the segmentation of the target regions and the latter shows the segmentation of the shadow regions. Although the MRF algorithm can detect and abstract both target and shadow regions, it is subject to the influence of background clutter, as seen, for example, in the segmentation of the SAR image shown in Fig. 4(A). Thus, the MRF algorithm performs poorly compared with the WD-CFAR algorithm.

**Figure 5:**
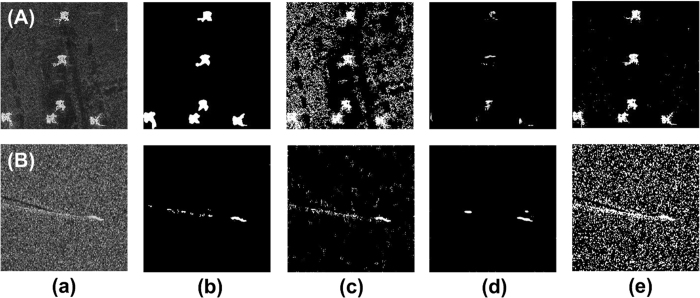
Segmentation results obtained using different algorithms for SAR images containing only target regions and no shadow regions. ((**a**) Original SAR images, (**b**) WD-CFAR algorithm, (**c**) CFAR method, (**d**) Wavelet transform, (**e**) MRF method.)

The next experiment differed from the two sets of experiments described above. Its purpose was to segment SAR images containing only target regions, with no shadow regions. The experimental results are shown in Fig. 5. These results were obtained under the same experimental conditions for the four algorithms. The relevant parameters for the images and the experiments are shown in Table 2. Note that the parameter *n*_F_ is used only in the WD-CFAR algorithm, the parameter *r* is only used in the WD-CFAR algorithm and the wavelet decomposition algorithm, and the constant false alarm rate parameter *P*_f_ is used in the WD-CFAR algorithm, the wavelet decomposition algorithm, and the direct CFAR method. Figure 5(a) shows the original SAR images. Figure 5(A) is an airborne SAR image with a size of 256 × 256. It was obtained from the Sandia Laboratory and contains multiple tank targets. The relevant characteristics of this image are that the shadow regions are not obvious or, at least, are not significantly different from the background clutter. This is a high-resolution and high-SNR image, with an SNR of 349.134, as shown in Table 2. Figure 5(B) shows SAR image data collected from space via ERS-2, with a size of 400 × 400. Figure 5(B) contains a single ship target. The SAR image shown in Fig. 5(B) contains no shadow region and has a low resolution and a low SNR. Figure 5(b) shows the segmentation results obtained using the WD-CFAR algorithm. As shown in Fig. 5(b), the segmentation effect is very good, and the target regions were effectively extracted.

The segmentation results of the CFAR algorithm are shown in Fig. 5(c). The false alarm rate for the CFAR algorithm was set identical to that for the WD-CFAR algorithm, as shown in Table 2. As shown in Fig. 5(c), the segmentation results are not very good. Similarly, when wavelet decomposition is applied for SAR image segmentation without the selection of particular coefficient sub-images to process, i.e., all sub-images are subjected first to detection and segmentation and then to inverse transform processing, it is very difficult to produce good results, as shown in Fig. 5(d). The reason for this is that all wavelet-decomposed sub-images are subjected to segmentation processing, which requires high contrast between the target and background regions to achieve good segmentation results. As shown in Fig. 5(e), the MRF segmentation algorithm can produce good segmentation results when applied to high-SNR SAR images, but it yields poor results on low-SNR SAR images.

### Quantitative analysis of algorithm performance

Above, the experimental results of the WD-CFAR algorithm, the direct CFAR detection algorithm, the direct wavelet transform algorithm, and the MRF algorithm were compared from a visual perspective. Next, specific evaluation factors were used for a further quantitative performance analysis. These evaluation parameters included the log-normalized likelihood ratio and mean of the ratio image, the target region segmentation ratio, and the run time of the algorithm. These parameters were used to describe and analyze different aspects of the performance of the four algorithms.

The term ratio image (RI) refers to the ratio of the segmented image to the original image, and its log-normalized likelihood ratio |*D*| is typically used to describe the heterogeneity of different regions of the image^27^. For the segmented image, a smaller value of |*D*| indicates smaller residual target structures in the segmented image, corresponding to a higher accuracy of target segmentation and extraction. The mean value *μ*_RI_ of the ratio image indicates how much information the image contains. If *μ*_RI_ is large, the image contains a large amount of information concerning the target region.

Let *I*_org_ (*i, j*) and *I*_seg_ (*i, j*) denote the original image and the segmented image, respectively. Then, the mathematical definition of the ratio image follows:


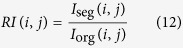


Here, *RI (i, j*) is the ratio image. If the size of the ratio image is *M* × *N*, then the definition of the mean value of the ratio image is expressed as shown in Equation (13):


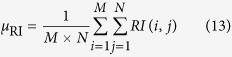


The log-normalized likelihood ratio of the ratio image is formulated as follows:


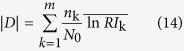


Here, *m* denotes the number of segmented regions of the same type that are obtained by segmenting an image, i.e., *m* separate sections that share uniformly homogenous characteristics. *n*_K_ is the number of pixels in the *k*th segmented uniform region, and 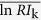 is the average value of the ratio image in the *k*th segmented uniform region. *N*_0_ denotes the total number of pixels, i.e., *N*_0_* = M* × *N*.

There are two types of target segmentation rates: true and false. The true segmentation rate is the probabilities that target pixels are segmented correctly, i.e., that pixels belonging to target regions are segmented as target pixels by the algorithm. The true segmentation rate is defined as follows:


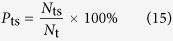


Here, *N*_ts_ denotes the number of pixels belonging to a target region that are found to belong to a target region by the algorithm and *N*_t_ denotes the total number of pixels in all target regions.

The false segmentation rate is the probability that pixels that do not belong to a target region are nevertheless identified as such by the segmentation algorithm. The false segmentation rate is defined as the ratio of the number of background clutter pixels identified as target pixels to the total number of pixels identified as target pixels:


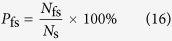


Here, *N*_fs_ is the number of pixels that belong to background clutter regions but are nevertheless identified as part of a target region. *N*_s_ is the total number of pixels identified as part of a target region by the algorithm: *N*_s_* = N*_ts_* + N*_fs_.

The run-time parameter *t* is an important index that can be used as a measure of the complexity of an algorithm. Because complex theories often give rise to complex algorithms, the corresponding run times will tend to be high.

**Figure 6:**
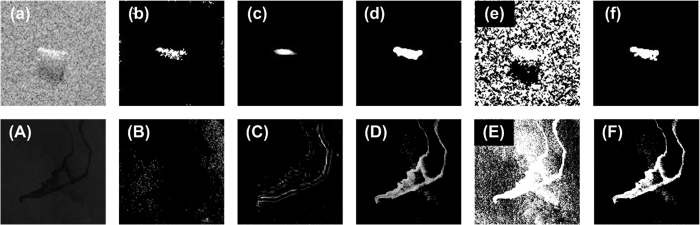
Experimental results used for quantitative analysis. ((a) and (**A**) original SAR images, (b) and (**B**) CFAR method, (c) and (**C**) wavelet method, (d) and (**D**) WD-CFAR algorithm, (e) and (**E**) MRF method, (f) and (**F**) ground truth.)

The experimental results are shown in Fig. 6 and Table 3. Here, Fig. 6(a,A) show the original images, and their parameters are listed in Table 3. The SAR image in Fig. 6(a) was obtained from the MSTAR database, and the target region contains tanks. Figure 6(A) shows SAR image data acquired from an ocean region by the European Space Agency’s ERS-2 satellite. The dark region in Fig. 6(A) is an oil-polluted belt, which was treated as the target region to be segmented in this experiment. The SAR images in both Fig. 6(a,A) have low SNR values. The segmentation results obtained using the CFAR detector are shown in Fig. 6(b,B). It is clear that the direct CFAR algorithm cannot be used to detect signs of oil pollution through the detection and segmentation of an oil-polluted band of ocean. The results presented in Fig. 6(c,C) were obtained using the wavelet transform theory. Although this method could detect the profiles of the target regions, the final effect was not good. The experimental results shown in Fig. 6(d,D) were obtained using the WD-CFAR algorithm. This algorithm was able to segment all target regions. The results of the MRF algorithm are shown in Fig. 6(e,E) and exhibit a poor segmentation effect. Figure 6(f,F) show the true target regions, which were obtained through visual interpretation. The experimental results presented in Fig. 6 further demonstrate that the WD-CFAR algorithm can effectively detect and segment weakly scattering target regions in SAR images, such as regions of oil pollution in the ocean.

Table 3 shows the various parameters used to quantitatively describe and compare the different algorithms, including the direct CFAR algorithm, the wavelet transform algorithm, the WD-CFAR algorithm, and the MRF algorithm, for experimental data shown in Fig. 6.

The same size and SNR parameters for the experimental images were used as inputs to all algorithms. Five parameters, i.e., |*D*|, *P*_ts_, *P*_fs_, *μ*_RI_, and *t*, were used as the quantitative evaluation indices for the different algorithms. Note that the parameter *P*_f_ is used in all algorithms except the MRF algorithm, whereas the parameter *r* is used in the WD-CFAR algorithm and the wavelet transform algorithm, and the parameter *n*_F_ is used only in the WD-CFAR algorithm. All parameter values are shown in Table 3. As shown, for the segmentation processing of the images shown in both Fig. 6(A,a), the minimum value of |*D*| was achieved by the WD-CFAR algorithm, which indicates that this algorithm demonstrates the best performance and the best segmentation effect in target region segmentation. The MRF algorithm produced the maximum value of |*D*|, reflecting its poor segmentation effect. For the SAR image shown in Fig. 6(a), the value of *P*_ts_ was the highest for the WD-CFAR algorithm, indicating that this algorithm achieved the most effective segmentation of the target region; for the image shown in Fig. 6(A), the *P*_ts_ value for the WD-CFAR algorithm is again higher than those for the wavelet transform algorithm and the direct CFAR algorithm but lower than that for the MRF algorithm. However, the *P*_fs_ value for the MRF algorithm is also very high for the SAR image in Fig. 6(A); the value of this quantity is 86.106%, far higher than that for the WD-CFAR algorithm, 17.036%. For the image in Fig. 6(A), the WD-CFAR algorithm achieved a lower *P*_fs_ than either the direct CFAR algorithm or the wavelet transform algorithm, indicating a lower rate of false segmentation. The mean *μ*_RI_ of the segmented target region image obtained using the WD-CFAR algorithm was higher than that achieved by any other algorithm except the MRF algorithm, indicating that this algorithm could obtain more target information than the other algorithms. The CFAR algorithm had the shortest run time, but its results were not good, and not all targets could be detected or segmented, as shown in Figs 4, 5 and 6. The run time of the WD-CFAR algorithm was typically shorter than that of the wavelet algorithm or the MRF algorithm. When all evaluation factors are considered together, the performance of the WD-CFAR algorithm is seen to be superior to that of the other three algorithms.

### Experiments on sliding window selection and computation of the optimal scale

**Figure 7:**
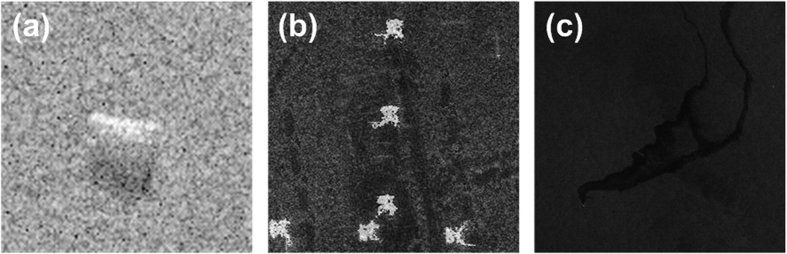
Experimental SAR images for determining the relationship between the optimal scale and the size of the sliding window.

The section that introduces wavelet decomposition theory mentions that the key step of wavelet decomposition is to determine the optimal scale *r*. When Equation (5) is used to compute the variable *LCV*_n_, a sliding window must be defined to obtain more accurate information. The size of the sliding window typically affects the calculation of the optimal scale. The purpose of this experiment was to investigate the effect of the window size on the optimal decomposition scale. The SAR images used in this experiment are shown in Fig. 7; the parameters related to these three SAR images have been introduced previously. The sizes of the images presented in Fig. 7(a–c) are 128 × 128, 256 × 256, and 1024 × 1024, respectively. The window size settings and experimental results are shown in Table 4.

As shown in Table 4, for each image, as the size of the sliding window increases, the value of the optimal scales for wavelet decomposition also slowly increases. The optimal scale *r*, however, remains relatively stable in a certain range. For example, for Fig. 7(a), the optimal scale is *r* = 3 for a sliding window size of 3 × 3, 5 × 5, or 7 × 7. Similarly, *r* = 4 is found for a window size of 9 × 9, 11 × 11, 13 × 13, or 15 × 15; however, when the size of the window is greater than 15 × 15, the optimal decomposition scale *r* remains unchanged, namely, *r* = 5. The images presented in Fig. 7(b,c) also exhibit similar behavior. This experiment shows that the size of the window has little influence on the selection of the optimal scale for wavelet decomposition. Another interesting problem is also evident from Table 4. When the sliding window size is 9 × 9 or 17 × 17, the optimal decomposition scale for the wavelet transform is the same for all three SAR images. However, in practical applications, the window size is generally 5 × 5 or 7 × 7. In the WD-CFAR algorithm, when computing the optimal decomposition scale *r* for all experiments, the size of the window was set to seven.

### Experiments on feature scale selection

**Figure 8:**
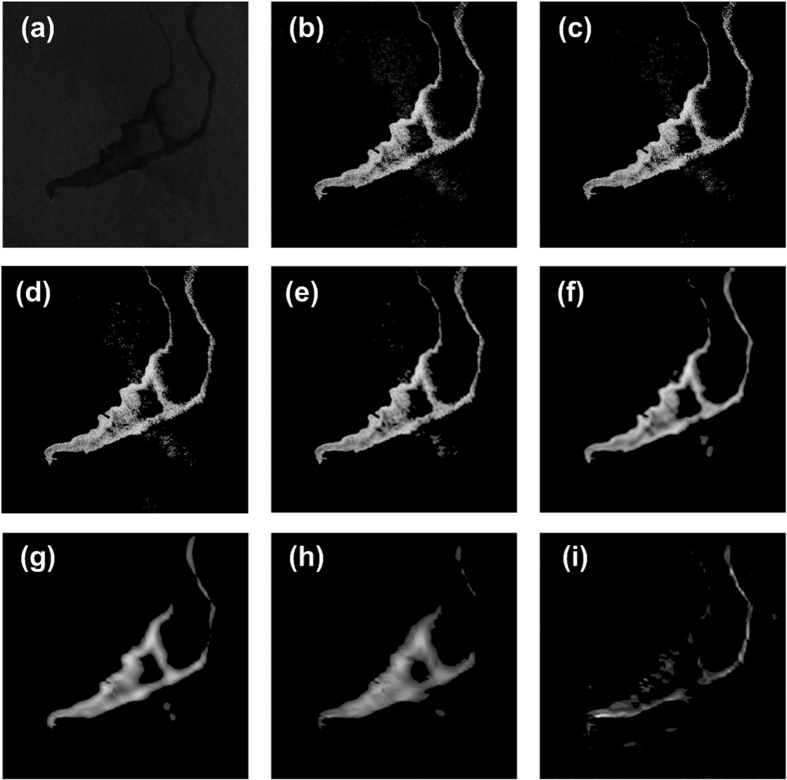
Target region segmentation results obtained using different feature scales *n*_F_. ((**a**) Original SAR image, (**b**) Ground truth, (**c**–**i**) Result for *n*_F_ = 1, 2, 3, 4, 5, 6, 7, respectively.)

In the implementation of the WD-CFAR algorithm as described above, the fourth step involves the selection of a subset of the coefficient sub-images. The following is a detailed experimental analysis of how the feature scale *n*_F_ for the coefficient sub-images should be chosen. As mentioned previously, the fundamental difference between the multi-scale wavelet decomposition segmentation algorithm and the WD-CFAR algorithm is that the WD-CFAR algorithm selects particular sub-images on which to perform the segmentation processing, whereas the wavelet transform algorithm not only does not select a subset of sub-images but in fact requires all sub-images to be subjected to detection and segmentation processing. SAR is coherent imaging radar, which inevitably produces considerable speckle noise; this poses severe problems in SAR image processing and interpretation. When multi-scale wavelet transform theory is used to decompose a SAR image, as the decomposition scale increases, the high-frequency information contained in the sub-images that are produced gradually decreases, while the proportion of background clutter components increases. When the objective is the target region segmentation of a SAR image, the target information is mainly concentrated in the low-scale sub-images, especially at the first and second scales. The high-scale sub-images mainly contain a large amount of background clutter information. If they are used to perform target region detection and segmentation, not only will they not produce good results, but they will also affect the target region segmentation. This experiment was performed to confirm the effect of sub-image selection; the experimental results are presented in Fig. 8 and Table 5.

Figure 8(a) shows the original SAR image, which is the same as that shown in Fig. 6(A). The segmentation goal was to extract the region polluted with oil, i.e., the dark region in the SAR image. The true segmented map of the oil-polluted region is shown in Fig. 8(b). In this experiment, when multi-scale wavelet theory was used to decompose the SAR image shown in Fig. 8(a), the preset decomposition scale was seven, i.e., *N* = 7, and the optimal decomposition scale *r* was found to be four for the sliding window size of 7 × 7, i.e., *r* = 4. This was computed using Equations (5, 6, 7). The segmentation results for the target region as the feature scale varies from the first scale to the seventh scale are shown in Fig. 8(c–i), respectively. When a feature scale *n*_F_ is selected, all coefficient sub-images at scales less than or equal to the feature scale are subjected to target detection and segmentation during the first round of CFAR detection in the WD-CFAR algorithm, whereas sub-images at scales higher than the feature scale are not processed. For example, if the feature scale is four, namely, *n*_F_ = 4, then all sub-images from the first scale to the fourth scale are subjected to segmentation, whereas sub-images at scales greater than four are not. As shown in Fig. 8, when the decomposition scale is greater than a certain value, if the scale is further increased, the segmentation results become worse and the error increases; in this case, the optimal scale was two, and when the scale was larger than two, the segmentation effect gradually degraded. For this reason, in the experiments using the WD-CFAR algorithm, the feature scale for target region segmentation was usually set to two, i.e., *n*_F_ = 2.

Table 5 shows the true and false segmentation rates achieved in the image segmentation process under different feature scale conditions using the WD-CFAR algorithm. As shown in Table 5, the difference in these rates was not large between the cases in which the feature scale was equal to one and two. To collect as much target information as possible, the feature scale was set to two, i.e., *n*_F_ = 2. Table 5 shows that when the feature scale is greater than four, the false segmentation rate begins to increase, which further suggests that the optimal decomposition scale in this experiment should be four, namely, *r* = 4, as found based on the theory introduced previously for determining the optimal scale *r*. As shown in Table 5, as the scale increases, the true segmentation rate gradually and continuously decreases, whereas the false segmentation rate initially decreases and then increases.

The results of the experiments described above show that the WD-CFAR algorithm is a suitable method for the segmentation of target and shadow regions in SAR images. The proposed algorithm not only does not require strong contrast in the SAR images to be subjected to detection and segmentation, namely, it is suitable for both high- and low-SNR SAR images, but it can also, by virtue of its use of two rounds of CFAR detection and its selection of a particular subset of the multi-scale wavelet decomposition coefficient sub-images for processing, reduce the influence of speckle noise and produce effective segmentation results.

The WD-CFAR algorithm is suitable for application to various types of SAR images, especially moderate- and low-resolution SAR images; in other words, it exhibits strong adaptability. It addition to target regions, it can also be used to detect and segment shadow regions, including weakly scattering or dark targets such as airport runways and bands of oil pollution in the ocean.

## Conclusions

Based on the characteristics of SAR images, CFAR detection theory, and the wavelet transform principle, this paper presents a new method of SAR image segmentation called the WD-CFAR algorithm. This method overcomes some of the disadvantages of CFAR detection theory and wavelet transform theory when applied individually. For example, it does not require strong image contrast for successful SAR image segmentation. It is also insensitive to speckle noise, and it can segment both target regions and shadow regions effectively, even weakly scattering targets such as airport runways. Verification experiments have shown that the WD-CFAR algorithm not only can perform successful SAR target and shadow segmentation but also can be applied to various types of SAR images. The next step in the further development of this algorithm will be to improve the segmentation accuracy and increase the amount of detail that can be collected. This may facilitate research on target and shadow region segmentation in high-resolution SAR images.

## Additional Information

**How to cite this article**: Huang, S. *et al*. A New SAR Image Segmentation Algorithm for the Detection of Target and Shadow Regions. *Sci. Rep.* **6**, 38596; doi: 10.1038/srep38596 (2016).

**Publisher's note:** Springer Nature remains neutral with regard to jurisdictional claims in published maps and institutional affiliations.

## Figures and Tables

**Table 1:** Parameter settings for the experiments presented in Fig. 3.

Image	Size	SNR	*r*(7 × 7)	*n*_F_	*P*_f_	
					First round	Second round
Figure 3(A)	128 × 128	57.269	3	2	10^−5^	10^−5^
Figure 3(B)	512 × 400	73.387	7	2	10^−5^	10^−10^
Figure 3(C)	400 × 400	81.798	7	2	10^−3^	10^−5^

**Table 2:** Parameter settings for the experiments presented in Fig. 5.

Image	Size	*SNR*	*r*(7 × 7)	*n*_F_	*P*_f_	
					First round	Second round
Figure 5(A)	256 × 256	349.134	3	2	10^−8^	10^−13^
Figure 5(B)	400 × 400	74.592	7	2	10^−8^	10^−13^

**Table 3:** Comparisons of performance parameters for different algorithms.

Image	Figure 6(a)				Figure 6(A)			
Size	128 × 128				1024 × 1024			
SNR	57.3186				64.6911			
**Method**	**CFAR**	**Wavelet**	**WD-CFAR**	**MRF**	**CFAR**	**Wavelet**	**WD-CFAR**	**MRF**
|*D*|	0.189	0.047	0.014	3.259	0.273	0.301	0.124	0.802
*P*_ts_	70.925	48.871	79.672	77.911	0.493	36.441	81.725	98.408
*P*_fs_	32.238	25.625	29.8373	0.031	99.471	56.373	17.036	86.106
*μ*_RI_	0.022	0.009	0.022	0.722	0.137	0.151	0.595	4.542
*t* (s)	0.335	0.984	0.549	1.313	1.881	20.225	10.525	4171.1
*r*	—	3		—	—	4		—
*n*_F_	—	—	2	—	—	—	2	—
*P*_f_	First round 10^−5^			—	First round 10^−1^			—
	Second round 10^−5^				Second round 10^−2^			

**Table 4:** Optimal scales for different window sizes.

Sliding window size		3×3	5×5	7×7	9×9	11×11	13×13	15×15
Optimal scale (*r*)	Figure 7(a)	3	3	3	4	4	4	4
	Figure 7(b)	2	3	3	4	4	5	5
	Figure 7(c)	3	4	4	4	5	5	5
**Sliding window size**		**17×17**	**19×19**	**25×25**	**35×35**	**45×45**	**65×65**	**105×105**
Optimal scale (*r*)	Figure 7(a)	5	5	5	5	5	5	5
	Figure 7(b)	5	5	6	6	7	7	7
	Figure 7(c)	5	6	6	6	7	7	7

**Table 5:** Segmentation rates at different feature scales *n*_F_.

Feature scale (*n*_F_)	1	2	3	4	5	6	7
True segmentation rate (*P*_ts_)	82.68	81.72	76.59	73.72	70.73	67.87	41.36
False segmentation rate (*P*_fs_)	18.76	17.04	13.18	**12.00**	12.69	19.32	18.69
